# Abnormalities of Sphingolipids Metabolic Pathways in the Pathogenesis of Psoriasis

**DOI:** 10.3390/metabo13020291

**Published:** 2023-02-16

**Authors:** Beatriz Burger, Roberta Nicolli Sagiorato, Isabella Cavenaghi, Hosana Gomes Rodrigues

**Affiliations:** Laboratório de Nutrientes e Reparo Tecidual, Faculdade de Ciências Aplicadas, Universidade Estadual de Campinas, Limeira 13484-350, SP, Brazil

**Keywords:** skin inflammation, ceramide, psoriasis

## Abstract

Psoriasis is immune-mediated skin disorder affecting thousands of people. Sphingolipids (SLs) are bioactive molecules present in the epidermis, involved in the following cellular processes: proliferation, differentiation, and apoptosis of keratinocytes. Alterations in SLs synthesis have been observed in psoriatic skin. To investigate if the imbalance in lipid skin metabolism could be related to psoriasis, we analyzed the gene expression in non-lesioned and lesioned skin of patients with psoriasis available in two datasets (GSE161683 and GSE136757) obtained from National Center for Biotechnology Information (NCBI). The differentially expressed genes (DEGs) were searched for using NCBI analysis, and Gene Ontology (GO) biological process analyses were performed using the Database of Annotation, Visualization, and Integrated Discovery (DAVID) platform. Venn diagrams were done with InteractiVenn tool and heatmaps were constructed using Morpheus software. We observed that the gene expression of cytoplasmic phospholipase A_2_ (*PLA2G4D*), glycerophosphodiester phosphodiesterase domain containing 3 (*GDP3*), arachidonate 12-lipoxygenase R type (*ALOX12B*), phospholipase B-like 1 (*PLBD1*), sphingomyelin phosphodiesterase 3 (*SMPD3*), ganglioside GM2 activator (*GM2A*), and serine palmitoyltransferase long chain subunit 2 (*SPTLC2*) was up-regulated in lesioned skin psoriasis when compared with the non-lesioned skin. These genes are related to lipid metabolism and more specifically to sphingolipids. So, in the present study, the role of sphingolipids in psoriasis pathogenesis is summarized. These genes could be used as prognostic biomarkers of psoriasis and could be targets for the treatment of patients who suffer from the disease.

## 1. Introduction

Skin is the largest organ in the human body and the surface most exposed to the external environment, acting as the first line of defense against external pathogens, as well as physical and chemical insults [1,2]. It is an organ divided into epidermis and dermis [3]. The epidermis is the topmost layer of skin and is composed mainly of keratinocytes (≈97%) distributed in the stratum corneum, granulosum, spinosum, and basal layers, distinguishing according to their degree of maturation and differentiation [4,5]. From the basal layer to the stratum corneum, keratinocytes are conditioned to cell division, differentiation, loss of nucleus, and death [6,7].

The dermis is rich in extracellular matrix and contains fibroblasts, endothelial cells, blood, and lymphatic vessels. In addition, dermis contains immune cells, such as neutrophils, macrophages, mast cells, dendritic cells, and lymphocytes. Since 1983, the skin is no longer considered just a passive physical barrier, it is now considered an immunologically active organ [8].

Immune responses in the skin are important for the body’s defense against pathogens. However, exacerbated immune reactions can cause chronic inflammatory skin diseases, such as psoriasis, which affects more than 125 million people worldwide [9,10,11,12]. This disease is more often found in adults than in children, with a bimodal distribution for disease onset with peaks between 30–39 years and 60–69 years. Moreover, there are no consistent results on significant differences by gender, affecting men and women equally [13,14].

Although data on the incidence and prevalence of psoriasis have increased in recent years, about 81% of the world’s countries lack epidemiological data [13]. There is still the need to improve the quality and standardization of data, especially in low- and middle-income countries, where there are greater gaps and underreporting [13].

The risk factors for psoriasis can be divided into two groups: extrinsic and intrinsic factors [15]. The extrinsic risk factors include mechanical stress, air pollution, drugs, vaccination, infection, consumption of alcohol, and smoking. Among the intrinsic factors, we found obesity, diabetes mellitus, dyslipidemia, hypertension, and mental stress [15,16,17]. All of these factors interact with the genetic background and can generate or exacerbate psoriasis [15].

Psoriasis also has gene-associated favoring factors and, heredity is the main risk factor for psoriasis development. This affirmation is supported by twin studies that have demonstrated a clear increase of two to three times more in monozygotic twins compared with dizygotic twins for developing psoriasis [18,19,20]. In a recent review, Dand and collaborators discussed the genetic basis of psoriasis and highlighted the contribution of HLA-C*06:02, a variant of the class I major histocompatibility complex (MHC-I) gene, as the main determinant of early psoriasis incidence [21,22]. The relevance of this variant was shown by studies that have demonstrated that the single nucleotide polymorphism of this allele generated the most powerful association with psoriasis in case-control studies [23,24]. Another important validated genomic region that is correlated with psoriasis is PSORS2. In this region, the gene CARD14, which encodes the nuclear factor kappa B activator, has been pointed out as a susceptible gene for common forms of psoriasis [25,26].

**Figure 1:**
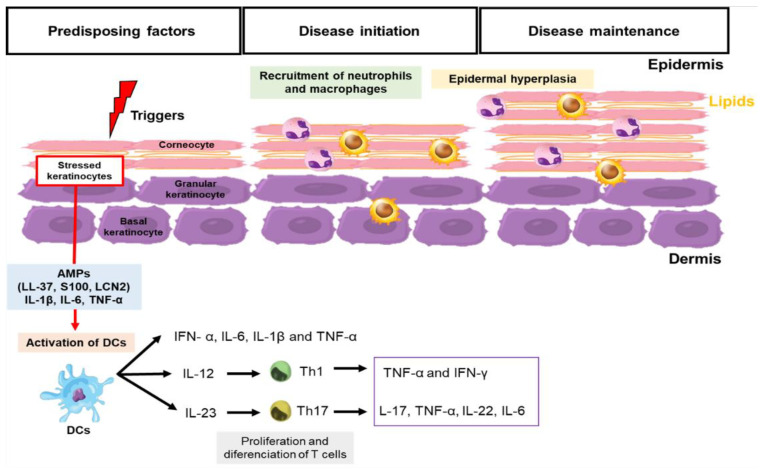
Pathophysiology of psoriasis. The pathophysiology of psoriasis involves excessive activation of the innate and adaptive immune system by extrinsic and intrinsic factors associated with the genetic background (triggers). Dendritic cells (DCs) are activated and secrete IFN-α, IL-6, IL-1β, and TNF-α. Then, dendritic cells induce the proliferation and differentiation of naive T cells. The activation of interleukin (IL)-12/Th1/interferon(IFN)-γ, and IL-23/Th17/IL-17 pathways lead to the induction and maintenance of skin inflammation and to an increase in the number of keratinocytes in the epidermis.

The orchestrated cascade of pathogenic events triggers psoriatic inflammation, activating keratinocytes, which become a source of antimicrobial peptides (AMPs) such as cathelicidins (LL-37), β-defensins, S100 family peptides, lipocalin 2 (LCN2), interleukins (IL)-1β, IL-6, and tumor necrosis factor alpha (TNF-α) (Figure 1) [27,28]. Such mediators induce innate immunity pathways, representing the first line of response for skin cells to the pathogenic environment of psoriasis (Figure 1) [27,28,29,30,31,32]. Then, the dermal dendritic cells are activated, producing IFN-α, IL-6, IL-1β, and TNF-α, and they migrate to the draining skin lymph nodes where they promote the differentiation of naive T cells into helper T cells—Th1 and Th17 (Figure 1) [1]. The stimulation of Th1 lymphocytes to secrete pro-inflammatory molecules, such TNF-α and IFN-γ, leads to the induction and maintenance of skin inflammation and increases the proliferation of keratinocytes in the epidermis. In turn, Th17 lymphocytes activated by IL-23 secretes effector cytokines, such as IL-17, TNF-α, IL-22, and IL-6 [32]. These cytokines lead to the inflow of neutrophils and other inflammatory cells to the skin, excessive proliferation of keratinocytes, and parakeratosis (Figure 1) [32,33,34,35,36].

The clinical presentation of psoriasis can differ, although it does express standard features such as inflammation characterized by erythema, thickening, and silvery/white scaly skin with variable distribution and severity [9,37]. The disease is known for its relapsing–remitting aspect. However, the trigger for remission remains unknown. About 80% to 90% of cases correspond to psoriasis vulgaris, also known as plaque psoriasis [9]. The other variants are less frequent and include guttate psoriasis, erythrodermic psoriasis, and pustular psoriasis [9]. Guttate psoriasis accounts for 2% of cases, usually occurs in children or adolescents, and is often triggered by streptococcal infections of the tonsils. Erythrodermic psoriasis is a severe variant of psoriasis that involves more than 75% of the body surface. Pustular forms of psoriasis are characterized by multiple sterile pustules and erythema, which can be localized on the palms and soles or in a generalized form [37,38].

Common regions affected by psoriasis vulgaris include the torso, extensor surfaces of the limbs, gluteal fold, and scalp, although any skin surface can be reached [38]. Psoriasis is not restricted to the skin as the presence of pro-inflammatory mediators is frequently reported in the circulation of patients. This characterizes psoriasis as a systemic inflammatory disease [38]. Consequently, it is often related to other comorbidities such as psoriatic arthritis, metabolic syndrome, and cardiovascular diseases [38,39]. In addition to the relationship with several comorbidities, psoriasis considerably affects psychosocial well-being, impairing the quality of life of most patients [37]. Understanding the pathophysiology of psoriasis is essential for the search for potential therapeutic targets.

Lipids are one of the fundamental components of skin (Figure 1). These hydrophobic molecules play a key role in the skin’s barrier function, inhibiting water loss and the entry of microorganisms, allergens, and other xenobiotics [3,40]. The outermost skin layer, the epidermis, is a lipid-rich region that provides structural support for skin [3]. The extracellular space of the epidermis is dominated by lipids, mostly sphingolipids, acylceramides, cholesterol, cholesterol esters, and non-esterified fatty acids (NEFA, commonly referred to as free fatty acids), arranged in multiple layers [3].

Sphingolipids are bioactive molecules, present in the human epidermis, involved in the maintenance of the skin barrier. The contribution of sphingolipids includes almost all aspects of molecular biology, having important functions in cell death, stimulating cell migration, inflammation, and cell differentiation [41,42,43,44]. Epidermal sphingolipids are formed from a sphingoid base with an amide-linked fatty acid and a polar head group that can be a carbohydrate (to form gangliosides, cerebrosides, and other glycolipids) or phosphorylcholine (to form sphingomyelins) [45,46]. Sphingolipids have distinct structures and functions; however, they are synthesized and degraded by convergent pathways.

Among all of the sphingolipids, ceramides are the most important sphingolipid present in the skin stratum corneum as it constitutes 50% of the epidermal lipids by mass [47]. Structurally, ceramide is composed of a sphingoid base linked with a fatty acid. Usually, four classes of fatty acids are found in ceramides: esterified ω-hydroxy (EO), ω-hydroxy (O), α-hydroxy (A), and non-hydroxy (N) fatty acids. These fatty acids can also differ by carbon chain length, which may result in different ceramides species [48] with distinct functions, mainly related to the water permeability barrier. A positive correlation between fatty acid length chain and improvement in skin barrier properties has been demonstrated [49,50,51]. A reduction in the ceramide fatty acid length chain induces modification in the lamellar structure periodicity, as well as lipid packing in the stratum corneum. These conditions are common in skin diseases [52]. Short-chain ceramides present a reduction in skin electrical impedance and an increase in skin permeability for indomethacin compared with long-to-medium ceramides [51,53]. Mechanistically, the excessive production of pro-inflammatory cytokines, mainly IFN-γ, inhibits the expression of elongase of long chain fatty acids (ELOVL) and ceramide synthase (CerS), resulting in the reduction in long-chain ceramides observed in psoriatic skin [54,55]. Thus, not only the amount of sphingolipid, but also its composition, is essential for stratum corneum barrier function.

**Figure 2:**
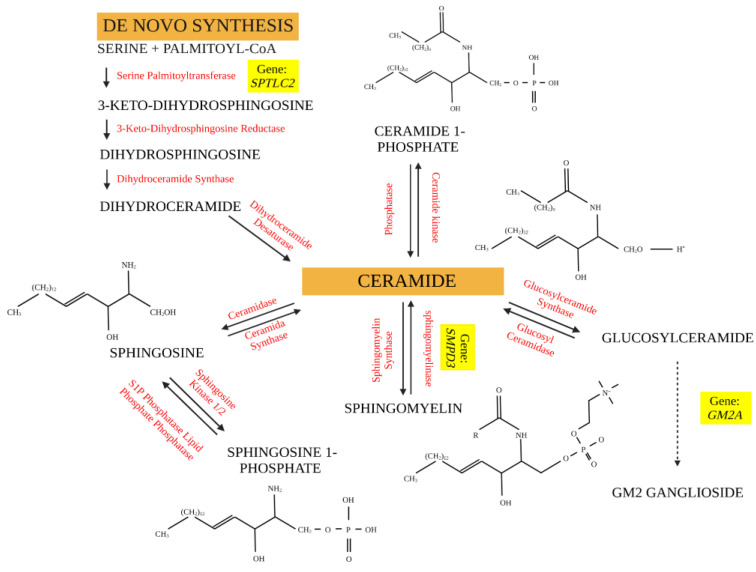
Sphingolipid metabolism. The synthesis of sphingolipids takes place in the endoplasmic reticulum, where serine and palmitoyl-CoA are condensed to form 3-ketodihydrosphingosine and are subsequently converted to ceramide. Ceramide gives rise to ceramide 1-phosphate, sphingosine, sphingosine 1-phosphate, sphingomyelin, and glucosylceramide. Genes highlighted in yellow are upregulated in the dataset analyzed and are directly involved in the biosynthesis of 3-ketodihydrosphingosine (SPTLC2), ceramide (SMPD3) and gangliosides (GM2A). The dotted arrow indicates the metabolic processes that precede the formation of GM2 gangliosides and are not relevant here.

In the endoplasmic reticulum (ER), de novo synthesis of sphingolipids takes place through the condensation of serine and palmitoyl-CoA to generate 3-ketodihydrosphingosine by the enzyme serine palmitoyltransferase (SPT). The 3-ketodihydrosphingosine molecule is reduced to dihydrosphingosine by NADPH-dependent 3-ketodihydrosphingosine reductase. Therefore, dihydrosphingosine is involved in the synthesis of all other subsequent sphingolipids, which initially occurs through the acylation of this molecule by a dihydroceramide synthase (CerS), which can be converted into a ceramide through the action of a desaturase (Figure 2). Ceramide, synthesized in the ER, is transported to the Golgi complex for subsequent conversion into sphingomyelin and glucosylceramide (precursor molecule of complex sphingolipids). In the plasma membrane, sphingomyelin is converted again into a ceramide that will be used in the synthesis of other sphingolipids, namely sphingosine, sphingosine-1-phosphate, and ceramide-1-phosphate [56,57,58] (Figure 2).

After de novo biosynthesis, ceramide is transported from the endoplasmic reticulum to the Golgi complex, where it can be converted into glycosphingolipids by ceramide glycosyltransferase or into sphingomyelin (SM) by sphingomyelin synthase (SMS1 and SMS2). Ceramide-derived sphingolipids will be stored in the plasma membrane and intracellular membranes [57]. After being allocated to the membrane, sphingomyelin can act in several cell signaling pathways or can be used as a substrate by sphingomyelinase for conversion into ceramide.

The composition of fatty acid chains present in the structure of sphingolipids may impact the cellular pathways and processes involved in the pathogenesis of diseases differently. Short-chain ceramides are freely transported by cytosol and positively regulate membrane permeability, while long-chain ceramides functionally contribute to the epidermal barrier, preventing water loss and the entry of xenobiotics [54,57]. The saturation of the head group or acyl chain and the chain length of these molecules influence interactions between proteins and lipids that are crucial for cell signaling and metabolism [53,57].

In addition to their epidermal barrier function, sphingolipids act in the signaling of cellular events that compromise the immune functions of the skin and, mainly, the proliferation, differentiation, and apoptosis of keratinocytes [45].

The interaction between keratinocytes, lipids, and sphingolipids guarantees the integrity of the epidermis and prevents excessive permeability to toxic molecules. Over the years, numerous studies have shown an association between the skin’s lipid composition and skin diseases, including psoriasis and atopic dermatitis.

Nomoto et al. (2018) identified that SMS2 deletion in a murine model resulted in a reduced glucosylceramide synthase activity, reduced sphingomyelin, CER[NS] and CER[AS] content in the stratum corneum, and increased skin permeability through transepidermal water loss. However, acanthosis, hyperkeratosis, parakeratosis, cell hyperproliferation and neutrophil infiltration were not observed in the skin of WT and SMS2 KO mice, indicating that reducing the SM content had little effect on the skin structure [59].

Sphingomyelin has been shown to be important for the development of regulatory T cells (Tregs), as acid sphingomyelinase (aSMase) deficiency in mice resulted in an increase in the number of Tregs in the spleen [60]. Tregs act critically against excess immunity to self-antigens and mediate self-tolerance and homeostasis. In psoriasis, Tregs are functionally defective and unable to control the exacerbated secretion of inflammatory cytokines [61].

The cerebroside and ganglioside complex glycosphingolipids are involved in the synthesis of biological membranes and undergo cyclic catabolism to form ceramides in the salvage pathway in lysosomes [62]. Glycosphingolipids are organized in the outermost layer of the cell membrane and act to maintain skin permeability; therefore, they may be involved in the development of skin diseases [63].

There are few studies that have evaluated the effects of gangliosides and cerebrosides in the context of psoriasis. Paller et al. (1993) investigated the effects of GM3 ganglioside on the in vitro proliferation of keratinocytes obtained from the skin of healthy patients and patients with psoriasis. GM3 treatment inhibited, in a dose-dependent manner, the proliferation of healthy and psoriatic keratinocytes, and this effect was not associated with cellular toxicity. The presented results suggest that the enzymes that synthesize and metabolize keratinocyte gangliosides and, thus regulate the concentration of these sphingolipids in the skin, participate in the regulation of proliferation by balancing the concentration of GM3 [64].

The expression pattern of CDw60, an acetylated form of the GD3 ganglioside, has been correlated with the stage of psoriasis. During the acute phase of the disease, there is little or no expression of CDw60, while there is a greater expression of this ganglioside in the basal layer keratinocytes in the chronic phase of psoriasis. CDw60 expression is stimulated in the presence of interleukin-4 (IL-4) or interleukin-13 (IL-13), both with a Th2 profile. Pretreatment with interferon-gamma (IFN-γ) blocked the action of IL-4 and IL-13 for CDw60 secretion. This effect appears to be related to the BCL-6 signaling pathway [65].

Considering the importance of lipids in skin barrier composition [3], emerging evidence also suggests the relevance of lipid metabolism in psoriasis [16,17,66]. As lipids are important constituents of the skin, we hypothesize that alterations in the skin lipid composition, especially in sphingolipids, may contribute to immune barrier dysfunction and immunological impairment, affecting psoriasis development.

## 2. Methodology

**Figure 3:**
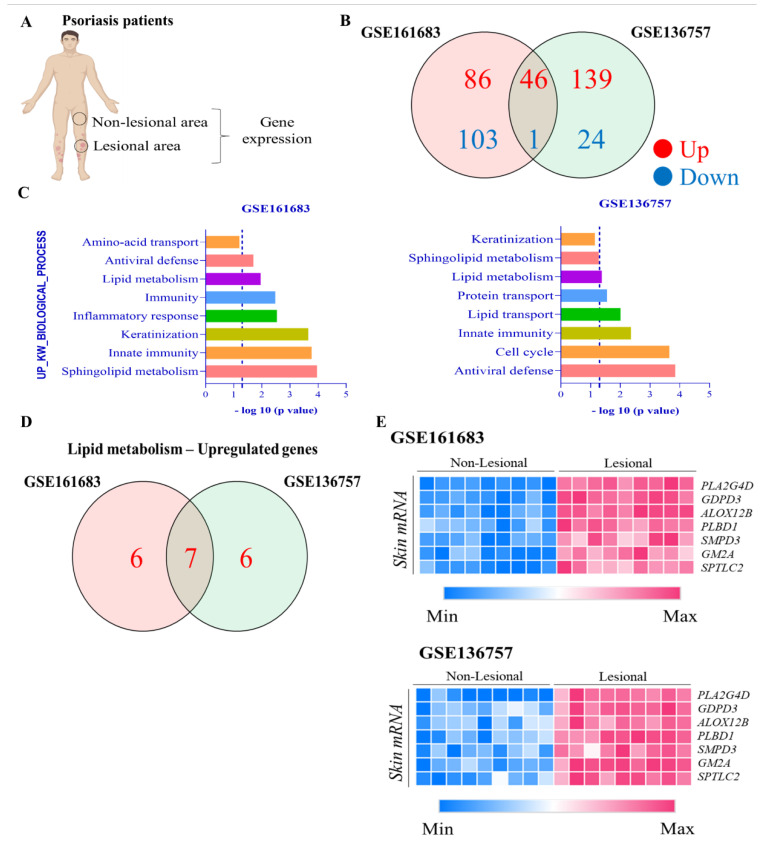
Importance of lipid metabolism in biological processes in psoriasis lesioned skin. (**A**) The gene expression as analyzed in non-lesioned and lesioned skin of patients with psoriasis from GSE161683 and GSE136757. (**B**) Venn diagram showing up- and down-regulated genes in the two datasets. (**C**) Up-regulated biological process. (**D**) Venn diagram showing up-regulated genes related to lipid metabolims in the two datasets. (**E**) Heat map showing expression gene profiles comparing non-lesional and lesional skin from the GSE161683 and GSE136757 datasets.

To investigate if the imbalance in lipid skin metabolism could be related to psoriasis, we analyzed the gene expression in non-lesioned (NP) and lesioned (LP) skin of patients with moderate-to-severe psoriasis (Figure 3A) available in two datasets (GSE161683 and GSE136757) obtained from the National Center for Biotechnology Information (NCBI).

In GSE161683, the researchers harvested NP and LP skin from nine patients with psoriasis. In addition, another nine patients from GSE136757 were analyzed. All of the gene expression profiling was analyzed using microarray analysis. The sample basic information of GSE161683 included the following: male and female patients with psoriasis vulgaris aged 33–56 years and PASI score of 12 [67]. For GSE136757, it included the following: male and female patients with plaque psoriasis aged 18–65 years with a PASI score of 12 [68].

In the present study, the differentially expressed genes (DEGs) were searched using GEO2R, an interactive web tool to identify genes that are differentially expressed across experimental conditions. This interface performs the comparisons on original submtter-supplied using GEOquery and limma R package from the Bioconductor Project. The enrichment analysis of DEGs was carried out by utilizing the online software Database of Annotation, Visualization, and Integrated Discovery (DAVID). The Gene Ontology (GO) biological process with a cut-off of *p* < 0.05 was chosen and transformed to −(Log10)*p*, which the value of −(Log10)*p* > 1.3 indicate the selected pathways. The Venn diagrams were done with InteractiVenn tool and heatmaps were constructed using Morpheus software.

## 3. Results

We observed that 46 genes were up-regulated, while 1 gene was down-regulated in these datasets analyzed (Figure 3B). The GO biological process analyses showed that up-regulated genes were related to lipid metabolism (Figure 3C). Among them, we observed 7 up-regulated genes in common in these datasets: cytoplasmic phospholipase A_2_ (PLA2G4D), glycerophosphodiester phosphodiesterase domain containing 3 (GDP3), arachidonate 12-lipoxygenase R type (ALOX12B), phospholipase B-like 1 (PLBD1), sphingomyelin phosphodiesterase 3 (SMPD3), monosialoganglioside2 activator (GM2A), and serine palmitoyl transferase long chain subunit 2 (SPTLC2) (Figure 3D). SMPD3, GM2A, and SPTLC2, genes related to sphingolipids metabolism, were up-regulated in lesioned skin psoriasis when compared with the non-lesioned skin (Figure 3E).

## 4. Discussion

Molecules and enzymes involved in sphingolipid metabolism may be differentially expressed in healthy controls and patients with mild or severe psoriasis [69]. Ceramides constitute the basis for the synthesis of sphingolipids, act as signaling molecules, regulate the differentiation of keratinocytes, and articulate immune responses [70]. The structural organization of the 18 different classes of ceramides depends on the chemical interaction between the fatty acids (non-hydroxy (N), α-hydroxy (AH), ω-hydroxy (O), and esterified ω-hydroxy (EO)) and the sphingoid base (dihydrosphingosine (DS), sphingosine (S), phytosphingosine (P), 6-hydroxysphingosine (H), and dihydroxysphinganine (T)) [71]. The normal epidermis is composed mainly of Cer[NH], Cer[AH], and Cer[NP], with acyl chain lengths ranging from C16 to C36, mostly composed of C24 [72].

Defects in the organization and composition of ceramides in the stratum corneum are related to the development and worsening of a psoriatic inflammatory condition. A recent study has shown that disruption of the epidermal barrier can exacerbate psoriasis by unregulated keratinocyte proliferation and the overexpression of S100A8, S100A9, CXCL1, and other molecules, indicating a defect in ceramide-dependent epidermal barrier signaling and function [73].

While severe psoriasis is associated with a higher concentration of sphingosine-1-phosphate (S1P), the distribution of ceramides is shown to be dysregulated concerning mild psoriasis and the healthy control, with a significantly reduced amount of this sphingolipid precursor resulting in greater transepidermal water loss [69,74]. Tawada et al. (2014) demonstrated a reduction in ceramides containing long-chain fatty acids in psoriatic lesions, which may be dependent on the reduction in the gene expression of ELOVL (long-chain fatty acid elongase) and ceramide synthase observed in the in vitro culture of keratinocytes [55]. This suggests that the lower concentration of ceramides is dependent on the reduction in the gene expression of enzymes that act in the elongation of fatty acid chains. Increased transepidermal water loss may be related to increased Cer[NS] and reduced Cer[NP], Cer[AP], and acylated ceramides [71].

In a recent review, Uchida and Park (2021) discussed the alterations in ceramide profile and skin disease associated with compromised permeability barrier functions. Among all of the sphingolipids, some studies have demonstrated a reduction in ceramide-containing phytosphingosine and esterified-omega-hydroxyacyl-sphingosine (EOS) in psoriatic skin compared with the control skin [75]. On the other hand, alpha-hydroxy-sphigosine (AS) and non-hydroxy-sphingosine (NS) are higher in psoriatic skin than in the control skin [75]. More recently, Checa and collaborators found an increase in the serum concentrations of sphingolipids in severe psoriatic patients compared with healthy controls [76]. Other studies also describe alterations in ceramides synthesis in psoriatic skin [77,78]. However, considering the complexity of sphingolipids synthesis with the consequent formation of different molecules, it is a challenge to simultaneously quantify every potential species.

As demonstrated in Figure 3E, the genes ALOX12B, PLA2G4D, and PLBD1 were up-regulated in psoriatic skin. These genes are involved in fatty acid metabolism mainly, but also play a role in the pathogenesis of psoriasis. In the present study, we present a resume of the main finds of these genes related to psoriasis. For more detailed information about fatty acid metabolism and psoriasis, we suggest considering Sorokin et al., 2018; Rioux et al., 2020; and Simard et al., 2022 [79,80,81].

The gene ALOX12B was up-regulated in lesional skin compared with non-lesional skin. The gene ALOX12B encodes the enzyme arachidonate 12-lipoxygenase R type (also known as 12(R)-LOX). 12(R)-LOX metabolizes linoleic acid to produce hydroperoxydecadienoic acid (9(R)-HpODE), a biologically active lipid mediator [82]. 9(R)-HpODE can be further metabolized to 9-hydroxy-octadecadienoic acid (9-HODE), or to hydroxy-epoxides such as 11-hydroxy-9,10-epoxy-linoleic acid (11H-9,10E-LA) and 13-hydroxy-9,10-epoxy-linoleic acid (13H-9,10E-LA) [83].

All of these metabolites, also known as oxylipins, have been found in inflamed skin and are related to inflammatory pain [83,84]. 12(R)-LOX seems to also play a crucial role in epidermal water barrier maintenance. Epidermal lipoxygenase-3 (eLOX3) together with 12(R)-LOX consecutively oxidize linoleic acid esterified in ω-hydroxy-ceramides. The free ω-hydroxyl released can bind to the proteins on the surface of corneocytes to form the corneocyte lipid envelope, a key component of the skin barrier [85]. 12(R)-LOX is also involved in the metabolization of arachidonic acid to 12-hydroxyeicosatetraenoic acid (12(R)HETE), which has been described as an important oxidized polyunsaturated fatty acid in psoriasis [85]. Psoriatic skin has an abundance of 12(R)HETE not found in non-lesional skin. From the current perspective, this excess of 12(R)HETE comes from the high expression of 12(R)-LOX together with the availability of arachidonic acid found in psoriasis [85]. The mutation in the ALOX12B gene is linked with the development of autosomal recessive congenital ichthyosis, confirming the importance of 12(R)-LOX for the maintenance of the skin permeability barrier [86].

Phospholipase A2 (PLA2) is a family of enzymes that play a critical role in the synthesis of lipid mediators via catalyzing the release of fatty acids from the cellular membrane [87]. Lipid mediators are involved in many physiologic processes such as inflammation, host defence, and barrier function [87]. The expression of a novel cytosolic PLA2, namely cPLA2δ or PLA2G4D, was observed in psoriatic lesions, yet was absent in healthy normal skin [88,89].

cPLA2 hydrolyses membrane phospholipids in the sn-2 position, releasing fatty acids such as arachidonic acid, which can be metabolized to eicosanoids, which are involved with inflammation. An association between high systemic PLA2 activity and metabolic syndrome in patients with psoriasis has been associated [90]. Cheung et al. (2016) showed that PLA2G4D is elevated in psoriatic mast cells and facilitates CD1a expression, which can be recognized by lipid-specific CD1a-reactive T cells, leading to the production of IL-22 and IL-17A [90]. The combination of tumour necrosis factor-alpha (TNF-α) and interleukin (IL)-17A is a strong inducer of PLA2G2F, PLA2G4D, and PLA2G4E expression, and these PLA2s play a major role in the proinflammatory effects of IL-17A and TNF-α on the epidermis, as pharmacologic inhibition or siRNA-mediated silencing of these enzymes leads to marked blunting of these cytokine responses in both inflammatory and differentiation-related processes [87].

Phospholipase B domain containing 1 is an enzyme with a phospholipase activity that, in humans, is encoded by the PLBD1 gene. PLBD1 is involved in phospholipid catabolic process, mainly processes related to glycerophospholipid biosynthesis [91]. Interestingly, not much is known about PLBD1 and skin disease/homeostasis. Only one study has described that the gene expression of PLBD1 is increased in psoriatic skin [91]. This gene was recently related to diverse types of cancer [92,93,94]. In 2020, a group demonstrated that PLBD1, together with eight other genes, can be a biomarker for the diagnosis of pancreatic adenocarcinoma [95].

Glycerophosphodiester phosphodiesterase domain-containing 3 (GDPD3) was also up-regulated in the two datasets evaluated. Once again, the role of these genes in skin metabolism is unknown. Glycerophosphodiesterases (GDE) are highly conserved enzymes from bacteria to mammals, which differ in their enzymatic characteristics and biological functions [96]. In humans, seven GDE isoforms (GDE1 to GDE-7) were described with high degree of tissue and functional specificity [96]. Although the first studies that described GDE in mammalian tissues date from 1956 [97], only at the beginning of the 20th century were GDE4, GDE6, and GDE7 characterized [98,99]. So, little information is actually available regarding their activity.

The gene glycerophosphodiester phosphodiesterase domain-containing 3 (GDPD3) encodes the glycerophosphodiesterase 7 (GDE7) isoform. Studies have shown that it is difficult to differentiate GDE4 from GDE7, as both isoforms present more than 50% amino acid identity, are located in the same region in cytosol, and present similar transmembrane organization [100]. Both isoforms present lysophospholipase D activity on lysophospholipids, but GDE7 produces lysophosphatidic acid (LPA) and cyclic phosphatidic acid (cPA), while GDE4 produces only LPA [100]. LPA is involved in multiple physiological and pathological processes in the skin. It not only regulates skin function, but also plays an important role in hair follicle development, skin wound healing, pruritus, skin tumors, and scleroderma [101]. Regarding psoriasis, Lei et al. (2021) described an increase in serum LPA concentrations in patients with psoriasis and in skin psoriatic lesions in the imiquimode (IMQ) mice model of psoriasis [101]. To investigate the effects of LPA on psoriasis, the authors treated IMQ mice with LPA topically and found an increase in epidermal thickness in the ears and aggravation of the PASI score (Psoriasis Area and Severity Index). Although LPA did not modulate Th1 and Th17 differentiation, it raised the expression of the inflammatory markers on psoriatic skin, suggesting an effect on keratinocytes. LPA also induced the activation of its receptor LPAR5 on the keratinocytes [101] and macrophages [102]. The activation of LPAR5 on immune cells was related with NLRP3 inflammasome activation during psoriasis development [102]. Other LPA receptors also play a role in the pathogenesis of psoriasis. LPAR1/3 inhibition (pharmacologically) alleviated skin symptoms in IMQ-induced psoriasis-like mouse models and decreased keratinocyte proliferation in the lesion. At the same time, LPAR1 knockdown in HaCaT cells reduced LPA-induced proliferation, suppressed cyclin A2 and CDK2 expression, and restored p27^Kip1^ expression [103].

Individuals with hepatic steatosis have an increased expression of GDPD3, which was related to the accumulation of triacylglycerol in the liver compared with healthy individuals [104]. GDPD3 has lysophospholipase D activity, which produces LysoPA from lysophospholipids in non-hepatic cells. LysoPA is an intermediate in the glycerol phosphate pathway for TAG biosynthesis [104]. In cancer research, disruption of the GDPD3 gene significantly decreased the self-renewal capacity in murine chronic myelogenous leukemia (CML) stem cells in vivo. This result suggests that lysophospholipid metabolism plays an important role in CML stem cells in vivo [105]. Although indirectly, these studies demonstrate a role for GDPD3 on psoriasis pathogenesis.

Although no actual information is available about the ganglioside GM2 activator (GM2A), it has previously been related to the AB variant of GM2 gangliosidosis. GM2A codifies the protein GM2 protein activator (GM2AP), which is an essential cofactor to β-hexosaminidase A in the degradation of GM2 to GM3. The absence/defect of GM2A seems to be the cause of GM2 accumulation in neuronal tissues in patients with GM2 gangliosidosis [106].

The level of ceramides in the epidermis results from the balance between the activities of ceramide-generating enzymes, such as serine palmitoyl transferase in the de novo synthesis pathway and sphingomyelinase, and the activities of degradative enzymes such as ceramidase [107].

Sphingomyelinase (nSMase) is a family of enzymes that hydrolyzes the membrane lipid sphingomyelin to generate phosphocholine and ceramide [108]. Until now, four types of nSMase have been described (nSMase1 to nSMase4) [109]. Among these isoforms, much attention has been given to nSMase2, as it is involved in diverse cell functions such as proliferation, cell death, apoptosis, and inflammatory responses, resulting in its involvement in the pathogenesis of several diseases such as cancer and psoriasis [110]. nSMase2 is encoded by the gene sphingomyelin phosphodiesterase 3 (SMPD3) and is localized in the plasma membrane and Golgi apparatus in different cell types [111,112].

nSMase2 is activated by stress factors such as oxygen and nitrogen reactive species; pro-inflammatory cytokines, mainly TNF-α and IFN-γ; and UV radiation, among others [110]. Activated keratinocytes release TNF-α, which binds to its receptor TNFR1. The activation of TNFR1 upregulates the nSMase2 activity, increasing the production of ceramide. In a positive loop, ceramide induces the translocation of the transcription factor NF-kB to the nucleus, where it promotes the expression of pro-inflammatory cytokines, such as TNF-α [113].

Regarding psoriasis, nSMase2 activity has been implicated in the increased production of metalloproteinase-9 (MMP9) in psoriatic keratinocytes [114]. MMP-9 is one of the regulators of keratinocyte proliferation, so it’s up-regulation is related to the excessive proliferation of keratinocyte, as observed during the pathogenesis of psoriasis. More recently, Chen et al. (2020) described the role of MMP-9 as a mediator of the cross-talk between neutrophils and endothelial cells during psoriasis. The authors demonstrated that MMP-9 is crucial for the vascular dysfunction observed in psoriatic skin [115]. On the other hand, a reduction in the levels of sphingomyelinase on the stratum corneum of lesional psoriatic skin compared with non-lesional skin has been described [116]. This finding has been corroborated by other study that demonstrated a diminished concentration of ceramides on the lesional epidermis of psoriatic patients in relation to the non-lesional epidermis, followed by a negative correlation between the ceramide content and PASI score [107]. The authors speculated that the nSMase activity could be reduced in these patients [107].

The discrepancies among the studies can be explained by the complexity of sphingolipid metabolism, as well as the differences in the skin region analyzed. As we know, the skin covering the foot is structurally different from facial skin, so the composition of sphingolipids should also be diverse [117]. Investigating 12 different anatomical skin sites, Merleev et al. (2022) found that 272 monitored lipids (from a list with 350) had anatomical specificity. In some cases, the same ceramide was increased in one anatomical site and reduced in another [117], illustrating the complexity of skin lipidomics.

Sphingomyelin phosphodiesterase 3 (SMPD3) is a cell membrane enzyme that hydrolyzes sphingomyelin to form ceramides and phosphocholine [118]. SMPD3 modulates sphingolipid metabolism through membrane trafficking, receptor clustering, and signal transduction. SMPD3 gene expression is dependent on inflammatory cytokines, such as TNF-α, interferon-gamma (IFN-γ), and IL-1β, and, consequently, activates caspase and calpain in a calcium-dependent manner [76]. Although the importance of ceramides in skin homeostasis is well documented, not much is known about the role of SMPD3 in skin metabolism. Up-regulation of SMPD3 has been described in the epidermis of knockout mice for the transcription factor MafB (MafB^−/−^ mice). The absence of MafB was related to impairment of epidermal keratinocyte differentiation [119]. On the other hand, using a non-invasive transcriptomic analysis of surface lipids, Shima and colleagues (2022) observed a lower expression of SMPD3 in children with mild-to-moderate atopic dermatitis compared with healthy children [120]. Atopic dermatitis, similar to psoriasis, is a multifactorial skin disease triggered by activation of adaptative immunity involving Th1, Th17, and Th22 cytokines pathways [121,122].

Another gene that is modulated in psoriatic skin is serine palmitoyltransferase long chain subunit 2 (SPTLC2). SPTLC2 codifies the enzyme serine palmitoyltransferase (SPT) that catalyzes the rate-limiting step in sphingolipids biosynthesis [117]. SPTLC2 has site-specific gene expression, and acts, preferentially, on long chain fatty acids at the sphingoid base, generating sphingolipids with more than 18 carbon atoms. SPTLC2 was up-regulated and SPTLC3 was down-regulated in the acral skin granular layer keratinocytes, illustrating the biogeographic expression pattern [117]. The authors explored a myriad of lipid profiles and observed diverse correlations among sphingolipids, skin regions, and skin diseases such as psoriasis and atopic dermatitis. Once again, it was demonstrated that the lipid expression in keratinocytes regulates their ability to respond to stress stimulus and to build up the immune response. On the other hand, the authors also observed that the psoriatic environment modulates the synthesis of sphingolipids. By characterizing the gene expression of 50 primary human keratinocyte cell lines under different culture conditions, it was apparent that in vitro culture with psoriasis-associated cytokines (TNF and IL-17A) increased the expression of SPTLC2, which matched the expression pattern of this gene in psoriasis lesional skin [117]. Other groups have already found increased expression of SPTLC2 in psoriatic skin [74,123] and demonstrated a correlation with SPTLC2 and trans epidermal water loss, reinforcing the relevance of sphingolipids for skin water barrier function.

## 5. Conclusions

In conclusion, we explored the alterations in the gene expression related to sphingolipid metabolism in psoriatic skin and demonstrated, based on the current literature, that sphingolipids play a role in the pathogenesis of psoriasis.
